# Supplementary material for: The Beneficial Effects of Triterpenic Acid and Acteoside in an In Vitro Model of Nonalcoholic Steatohepatitis (NASH)
Source: Int J Mol Sci. 2022 Mar 24;23(7):3562. doi: 10.3390/ijms23073562 (PMC8998673; doi:10.3390/ijms23073562)
Supplement: Supplementary file 1 [file ijms-23-03562-s001.zip › ijms-1619997-supplementary.pdf]

Supplementary Material

The beneficial effects of Triterpenic acid and Acteoside in an *in vitro* model of Nonalcoholic steatohepatitis (NASH)

Noel Salvoza[1,2], Chiara Bedin[3], Andrea Saccani[3], Claudio Tribelli[1], and Natalia Rosso[1]*

[1] Fondazione Italiana Fegato – ONLUS Area Science Park Basovizza SS14 km 163.5 34149 Trieste, Italy
[2] Philippine Council for Health Research and Development, DOST-Bicutan Taguig City 1631 Philippines
[3] ABRESEARCH SRL, via dell'Impresa, 1, 36040 Brendola (VI), Italy
* Correspondence: natalia.rosso@fegato.it

Number of supplementary figures: 5

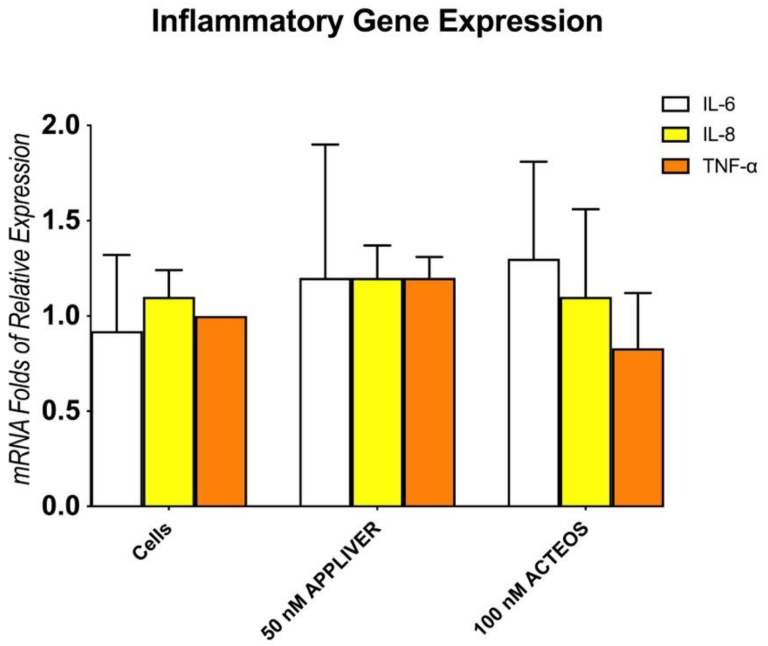

Figure S1. Assessment of anti-inflammatory properties of APPLIVER and ACTEOS without FFA on inflammatory cytokines mRNA expression. Gene expression analysis of pro-inflammatory cytokines (folds of mRNA expression *vs* cells without treatment). Values presented are the mean ± SD of three independent experiments. No significant results *vs* cells. Abbreviations: FFA, free fatty acids; IL-6, interleukin-6; IL-8, interleukin-8; TNF-α, tumor necrosis – alpha.

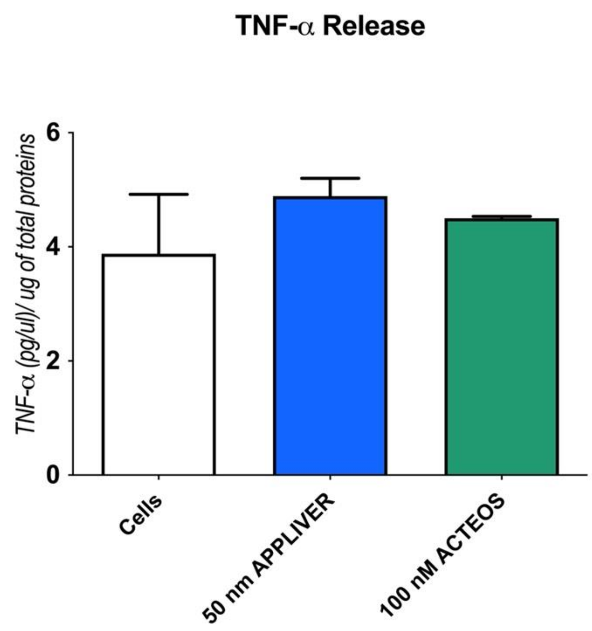

Figure S2. Quantification of TNF-α release in cell culture supernatant ((pg/μl)/ μg of total proteins) upon treatment of either APPLIVER (A) or ACTEOS (B) without FFA after 1h. Values presented are the mean ± SD of three independent experiments. No significant results *vs* cells. Abbreviations: FFA, free fatty acids; TNF-α, tumor necrosis – alpha.

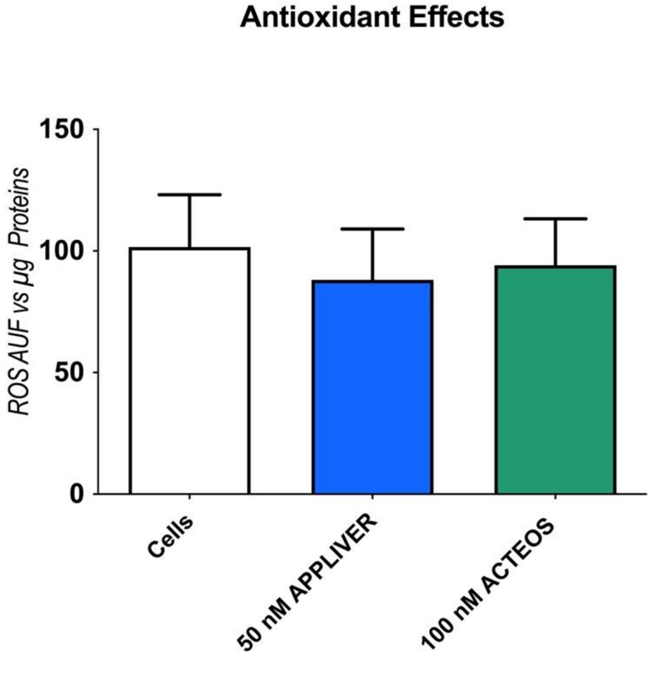

Figure S3. Assessment of antioxidant properties of APPLIVER and ACTEOS without FFA. Fluorescence was normalized by the total proteins present in the cell lysates (μg) assessed using fluorescamine assay. Values presented are the mean ± SD of three independent experiments. No significant results *vs* cells. Abbreviations: AUF, arbitrary unit of fluorescence; FFA, free fatty acids; H_2O_2, hydrogen peroxide.

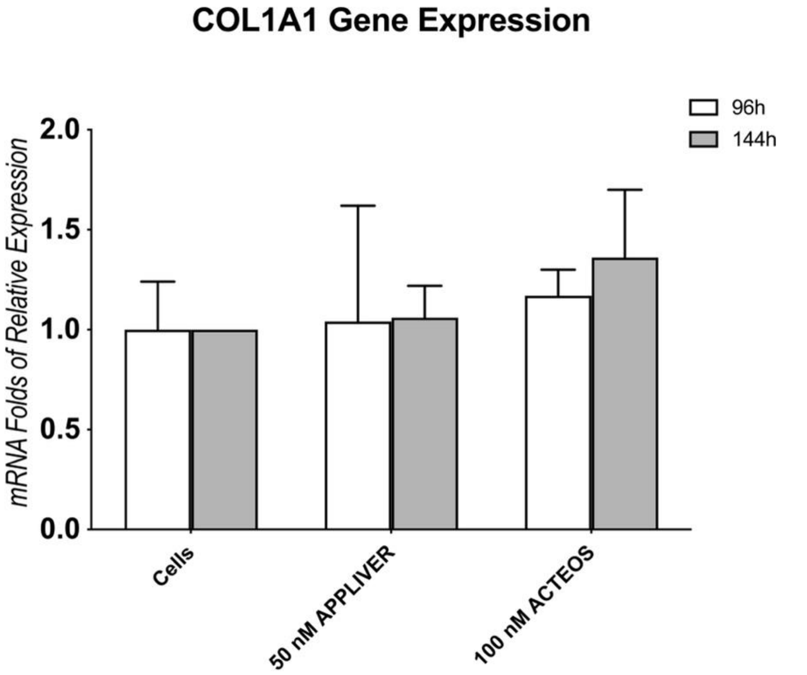

Figure S4. Gene expression of COL1A1 without exposure to FFA and APPLIVER and ACTEOS after 96h and 144h. Values presented are the mean ± SD of three independent experiments. No significant results *vs* cells. Abbreviations: COL1A1, collagen type I alpha 1 chain; FFA, free fatty acids.

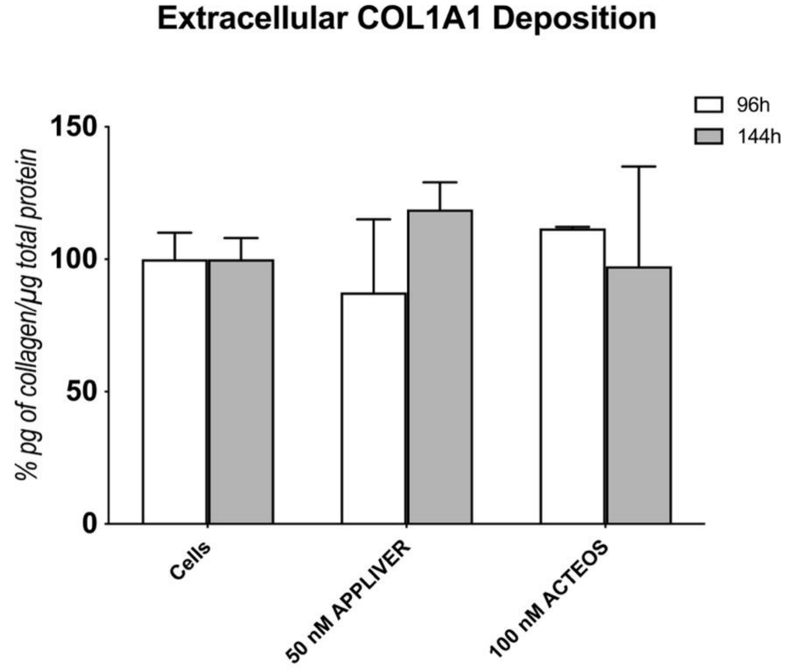

Figure S5. Quantification of extracellular COL1A1 deposition (% pg of collagen/µg total protein) using ELISA upon treatment of either APPLIVER or ACTEOS ± FFA after 96h and 144h. COL1A1 deposition was normalized to the total proteins in the cell lysate and reported as percentage *vs* Vehicle. Values presented are the mean ± SD of three independent experiments. No significant results *vs* cells. Abbreviations: COL1A1, collagen type I alpha 1 chain; FFA, free fatty acids.
